# Social Jetlag and Prostate Cancer Incidence in Alberta’s Tomorrow Project: A Prospective Cohort Study

**DOI:** 10.3390/cancers12123873

**Published:** 2020-12-21

**Authors:** Liang Hu, Andrew Harper, Emily Heer, Jessica McNeil, Chao Cao, Yikyung Park, Kevin Martell, Geoffrey Gotto, Grace Shen-Tu, Cheryl Peters, Darren Brenner, Lin Yang

**Affiliations:** 1 Department of Sport and Exercise Science, Zhejiang University, Hangzhou 310028, China; lianghu@zju.edu.cn; 2 Cancer Epidemiology and Prevention Research, Cancer Care Alberta, Alberta Health Services, Calgary, AB T2S 3C3, Canada; Andrew.Harper@albertahealthservices.ca (A.H.); Emily.Heer@albertahealthservices.ca (E.H.); Jessica.McNeil2@albertahealthservices.ca (J.M.); Cheryl.Peters@albertahealthservices.ca (C.P.); 3 Department of Surgery, Washington University School of Medicine, St. Louis, MO 63110, USA; caochao@wustl.edu; 4 Program of Physical Therapy, Washington University School of Medicine, St. Louis, MO 63110, USA; yikyungpark@wustl.edu; 5 Department of Oncology, University of Calgary, Calgary, AB T2N 4N2, Canada; Kevin.Martell@albertahealthservices.ca (K.M.); Darren.Brenner@ucalgary.ca (D.B.); 6 Department of Surgery, University of Calgary, Calgary, AB T2N 4N2, Canada; Geoffrey.Gotto@albertahealthservices.ca; 7 Alberta’s Tomorrow Project, Cancer Care Alberta, Alberta Health Services, Calgary, AB T2T 5C7, Canada; Grace.ShenTu@albertahealthservices.ca; 8 Department of Community Health Sciences, University of Calgary, Calgary, AB T2N 1N4, Canada

**Keywords:** social jetlag, circadian disruption, prostate cancer incidence

## Abstract

**Simple Summary:**

Due to social obligations (e.g., school, work), people shift their sleep and activity time regardless of their sleep-wake preference. To compensate for the lack of sleep accumulated over the workdays, people tend to oversleep on a work-free day. This difference in sleep timing between workdays and free days resembles traveling across different time zones, which causes jetlag (a mild form of circadian disruption). Thus, it is named social jetlag. Social jetlag has been linked with obesity, metabolic disorders, and cardiovascular risk in previous research. This study assessed social jetlag in 7455 cancer-free men in Alberta’s Tomorrow Project and followed them for on average 9.6 years, 250 men were diagnosed with prostate cancer. The study found that the more social jetlag men experienced, the greater their prostate cancer risk was. This finding warrants future research to better understand the complex behavioral and biological pathways between social jetlag and prostate cancer risk.

**Abstract:**

We investigated the association of social jetlag (misalignment between the internal clock and socially required timing of activities) and prostate cancer incidence in a prospective cohort in Alberta, Canada. Data were collected from 7455 cancer-free men aged 35–69 years enrolled in Alberta’s Tomorrow Project (ATP) from 2001–2007. In the 2008 survey, participants reported usual bed- and wake-times on weekdays and weekend days. Social jetlag was defined as the absolute difference in waking time between weekday and weekend days, and was categorized into three groups: 0–<1 h (from 0 to anything smaller than 1), 1–<2 h (from 1 to anything smaller than 2), and 2+ h. ATP facilitated data linkage with the Alberta Cancer Registry in June 2018 to determine incident prostate cancer cases (*n* = 250). Hazard ratios (HR) were estimated using Cox proportional hazards regressions, adjusting for a range of covariates. Median follow-up was 9.57 years, yielding 68,499 person-years. Baseline presence of social jetlag of 1–<2 h (HR = 1.52, 95% CI: 1.10 to 2.01), and 2+ hours (HR = 1.69, 95% CI: 1.15 to 2.46) were associated with increased prostate cancer risk vs. those reporting no social jetlag (*p* for trend = 0.004). These associations remained after adjusting for sleep duration (*p* for trend = 0.006). With respect to chronotype, the association between social jetlag and prostate cancer risk remained significant in men with early chronotypes (*p* for trend = 0.003) but attenuated to null in men with intermediate (*p* for trend = 0.150) or late chronotype (*p* for trend = 0.381). Our findings suggest that greater than one hour of habitual social jetlag is associated with an increased risk of prostate cancer. Longitudinal studies with repeated measures of social jetlag and large samples with sufficient advanced prostate cancer cases are needed to confirm these findings.

## 1. Introduction

Prostate cancer is the second most frequently diagnosed cancer, as well as the second-highest cause of cancer death among men globally [1]. However, the etiology of prostate cancer is poorly understood with very few risk factors identified thus far. Increasing age, African descent, and genetic predisposition have been suggested to increase the risk of developing prostate cancer [2]. Modifiable risk factors are not well known, but may include dietary components [2], selected occupational exposures [3], physical activity, and body composition (particularly visceral fat and thigh subcutaneous fat) [4,5]. Another factor that has been consistently associated with prostate cancer risk is circadian disruption, such as overly short or long sleep durations [6], late sleep timing [7], night shift work [7,8], and light cycle alteration [9]. However, social jetlag, a highly prevalent form of chronic circadian disruption [10], has been understudied in relation to prostate cancer risk.

Social jetlag refers to the misalignment between the internal clock and the socially required timing of activities [11] and can be operationally defined as the phenomenon that many individuals travel between socially imposed schedules on workdays and non-obligatory arrangements during free days [12]. Social jetlag resembles a mild yet chronic form of night shift work [13] and has been associated with obesity [10], depression [14], metabolic disorder [15,16], and adverse endocrine, behavioral, and cardiovascular risk profiles [17]. Similar to other forms of circadian disruption, social jetlag can misalign internal biological time organization with social timing constraints, resulting in unhealthy lifestyle behaviors such as smoking and possible alternations of the metabolic pathways [11].

Previous research suggested that social jetlag interacts with individual chronotype, the inter-individual differences in the phase of entrainment to the light-dark cycle [18], which may affect cancer risk. Having a late chronotype is associated with a greater risk of developing cancer compared to having an early chronotype, possibly due to more exposure to light at night and suppression of melatonin secretion [7,19]. Given that chronotype and desynchronization in sleep rhythm interact, previous studies included both circadian disruption and chronotype when assessing behavioral and clinical outcomes [7]. Papantoniou et al. [7] also found that the elevated prostate cancer risk among night shift workers was strongest for evening chronotypes, as well as morning chronotypes after long-term night work, suggesting an effect modification of chronotype. However, it is not clear whether this modification by chronotype exists for the association between social jetlag and prostate cancer risk.

To address these knowledge gaps, we prospectively investigated the association between social jetlag and prostate cancer incidence, and the potential effect modification of chronotype on this association in the Alberta’s Tomorrow Project (ATP) cohort [20].

## 2. Materials and Methods

### 2.1. Study Design and Population

The design, recruitment, enrolment, and data collection of the ATP cohort have been detailed elsewhere [21]. In brief, a total of 55,530 Albertans aged 35–69 with no personal history of cancer (other than non-melanoma skin cancer) were enrolled in a prospective cohort study between 2001 and 2015. Participants enrolled between 2001 and 2007 completed a survey in 2008 and constituted as the study baseline. Data were extracted on cancer-free men in the ATP cohort. All participants provided informed consent that their survey data could be used for research purposes and ethical approval was obtained from the Health Research Ethics Board of Alberta—Cancer Committee (HREBA.CC-19-0151 approved on 14 May 2019).

### 2.2. Ascertainment of Prostate Cancer

Participants were linked to the Alberta Cancer Registry with personal health numbers and followed to establish new cancer diagnoses. The Alberta Cancer Registry is a population-based registry, to which all physicians and laboratories in Alberta are mandated to report cancer diagnoses. The last data linkage occurred in June 2018.

### 2.3. Social Jetlag and Chronotype

In the 2008 survey (baseline), participants were asked to report their usual bed- and wake-time on weekday and weekend days in AM/PM, hour and minutes, respectively using the following questions: “On average over the past 7 days, at what time did you normally go to sleep on a weekday?”; “On average over the past 7 days, at what time did you normally wake up on a weekday?”; “On average over the past 7 days, at what time did you normally go to sleep on a weekend day?’ and “On average over the past 7 days, at what time did you normally wake up on a weekend day?”. In total, 7455 male Albertans who were cancer-free at the time of survey completion provided responses on the bed- and wake-times for the present study. Social jetlag was defined as the absolute difference in wake times between weekend days and weekdays [22]. Social jetlag was categorized into three groups by a one-hour increment in accordance with previous studies: 0–<1 h (45.6% of the sample population), 1–<2 h (29.6%), and 2+ hours (24.8%) [23,24]. Chronotype was defined by the tertiles of the sleep-time midpoint on free days (wake-time −½ total sleep duration), and categorized as an intermediate, early, or late sleep timing midpoint [6].

### 2.4. Covariates

A range of covariates were selected based on prior knowledge [25] including participant age at baseline, total household income (CAD $0 to $49,999; $50,000 to $99,999; ≥ $100,000), employment status (employed; unemployed), marital status (married, or living with a partner; divorced, separated, or widowed; single, never married), family history of cancer (yes; no), body mass index (BMI, measured in kg/m^2^) (normal/underweight: <25; overweight: 25 to <30; obese: 30+), smoking behavior (never smoker; former smoker; current smoker), drinking behavior (never drinker; rarely drinker; occasional drinker; regular drinker), the highest level of attained education (at least some post-secondary; the secondary school or less), history of any medical condition (yes; no), recreational physical activity (zero; non-zeroes grouped into quartiles), total sitting time (minutes/week), daily caloric consumption (measured in kcal/day), and sleep duration. In addition, we included information on their participation in prostate-specific antigen (PSA) testing prior to diagnosis (yes; no) to control for confounding by screening history [26].

### 2.5. Statistical Analyses

Participants’ follow-up time was calculated from their entry into the study (based on their exact age when competing the study survey in 2008. For more details, visit the Alberta ’s Tomorrow Project website: https://myatp.ca/resources/previous-surveys) to the date of cancer diagnosis (based on their exact age when their incident prostate cancer was diagnosed) or to the date of linkage with the Alberta Cancer Registry (based on their exact age at the time of data linkage). Subsequently, each participant in the study contributed person-time equivalent to the time between completion of Survey 2008 to their diagnosis, or to the time of linkage if diagnosis did not occur.

Descriptive analyses were conducted to assess participants’ characteristics according to the social jetlag group (0–<1, 1–<2, and 2+ h) using *t*-tests and chi-square tests as appropriate. Means (standard deviations) were calculated for continuous variables, and frequency percentages were calculated for categorical variables. A directed acyclic graph (DAG) was used to identify potential confounders a priori (Figure S1) including the following: age, marital status, the highest level of completed education, total household income, employment status, smoking status, frequency of alcohol consumption, recreational physical activity, total sitting time, pre-existence of medical conditions, and chronotype. Cox proportional hazards regression models were used to estimate the age-adjusted and multivariable-adjusted hazard ratios (HR) between categories of social jetlag (using the zero social jetlag group as the reference group) and 95% confidence intervals (95% CI) for prostate cancer incidence. Trends in social jetlag were assessed using the median value of each category of social jetlag (i.e., 0.5 for 0–<1 h; 1.5 for 1–<3 h; 2.5 for 2+ h). In sensitivity analyses, the multivariable-adjusted models were additionally adjusted for sleep duration and then adjusted for body mass index, daily caloric consumption, family history of cancer, and PSA testing. All statistical analyses were performed using Stata (v16.0). All statistical tests were 2-sided and statistical significance was set at *p* < 0.05.

## 3. Results

Between baseline assessment and follow-up, there were a total of 250 incident prostate cancer cases observed in the sample population of 7455 men enrolled in the ATP cohort. With a median follow-up of 9.57 years, 68,499 person-years were available for analysis. Table 1 presents the baseline sociodemographic characteristics, lifestyle factors, and screening behavior of this sample population, stratified by social jetlag category (0–<1 h, 1–<2 h, and 2+ h).

**Figure 1:**
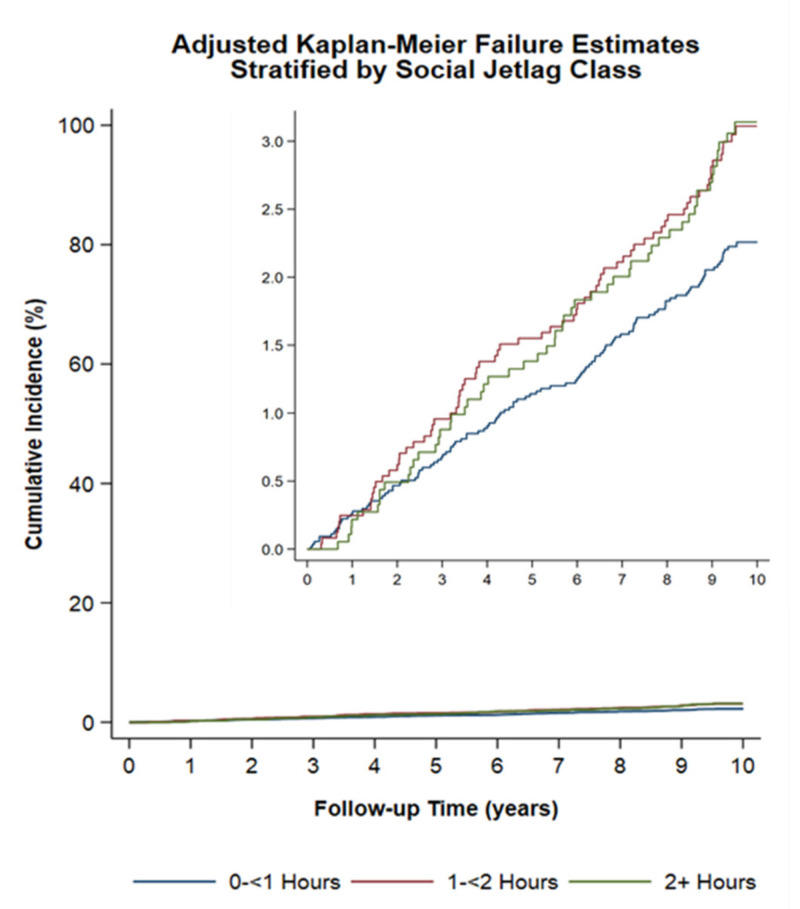
Prostate cancer incidence by social jetlag categories in the Alberta’s Tomorrow Project study (Adjusted for age, total household income, employment status, marital status, smoking status, frequency of alcohol consumption, the highest level of completed education, recreational physical activity, sitting time per week, pre-existence of medical conditions, and chronotype).

Table 2 shows the results for the age-adjusted and multivariable-adjusted HRs of prostate cancer incidence for “usual” social jetlag, both overall and in each chronotype subgroup. Men who experienced more than one-hour social jetlag (1–<2 h and 2+ h) had higher risks of prostate cancer than those in the lowest social jetlag category (0–<1 h). These association were present in the age-adjusted (1–<2 h: HR = 1.48, 95% CI: 1.09 to 2.00; 2+ hours: HR = 1.58; 95% CI: 1.12 to 2.22; *p* for trend = 0.004), multivariable-adjusted (1–<2 h: HR = 1.52, 95% CI: 1.10 to 2.01; 2+ hours: HR = 1.69; 95% CI: 1.15 to 2.46; *p* for trend = 0.004, Figure 1), sleep duration adjusted (1–<2 h: HR = 1.49; 95% CI: 1.09 to 2.06; 2+ hours: HR = 1.64, 95% CI: 1.12 to 2.41; *p* for trend = 0.006), and multivariable-adjusted models in sensitivity analyses (1–<2 h: HR = 1.45; 95% CI: 1.05 to 2.01; 2+ h: HR = 1.54; 95% CI: 1.04 to 2.27; *p* for trend = 0.018). When modeling with each of the chronotype subgroup, the increased prostate cancer risk associated with greater social jetlag remained significant among early chronotypes in the age-adjusted (1–<2 h: HR = 1.66; 95% CI: 1.08 to 2.54; 2+ hours: HR = 1.98, 95%C CI: 1.98, 95% CI: 1.11 to 3.52; *p* for trend = 0.04), multivariable-adjusted (1–<2 h: HR = 1.73, 95% CI: 1.12 to 2.68; 2+ hours: HR = 2.10, 95% CI: 1.16 to 3.77; *p* for trend = 0.003), sleep duration-adjusted (1–<2 h: HR = 1.75, 95% CI: 1.12 to 2.72; 2+ hours: HR = 2.12, 95% CI: 1.18 to 3.83; *p* for trend = 0.003), and multivariable-adjusted models in sensitivity analyses (1–<2 h: HR = 1.76; 95% CI: 1.13 to 2.75; 2+ hours: HR = 2.04, 95% CI: 1.11 to 3.74; *p* = 0.004) models.

## 4. Discussion

To our knowledge, this investigation is among the first to demonstrate that social jetlag is associated with an increased risk of prostate cancer. Drawing data from a large prospective cohort in Alberta, Canada, both multivariable- and latency multivariable-adjusted models suggest that habitual social jetlag greater than one hour may lead to a greater likelihood of developing prostate cancer compared to no social jetlag. In addition, these associations may be modified by chronotype. Men with early, but not intermediate or late, chronotypes may be exposed to an elevated risk of prostate cancer.

Increased prostate cancer risk has been associated with several markers of circadian disruption, such as night shift work [8], living in longitude positions of delayed solar time (i.e., further west) in a time zone [27], exposure to light at night [28], and sleep loss [29]. These negative health consequences are often attributed to the discrepancy between an individual’s biological circadian clock and social activities. Even though social jetlag is a prevalent form of circadian disruption, affecting 87% of the working population [13], its role in the etiology of prostate cancer is vastly understudied.

Shift work has been the center of research attempting to unravel the link between circadian disruption and cancer incidence and, in many ways, social jetlag resembles a mild form of shift work. Most studies repeatedly suggest that night shift work favors oncogenesis [30] and particularly increases the risk of breast, colorectal, and prostate cancer [7,8,31]. Night shift work presents a radical disturbance to internal biological rhythm and is often accompanied by sleep deprivation, as a person often finds it difficult to sleep when the internal circadian clock operates in waking mode regardless of being tired after night shift work. This disturbance is proposed to be the cause of some health consequences including elevated prostate cancer risk associated with rotating shift work [32].

In contrast, social jetlag is not necessarily a sleep-depriving behavior. Shifting sleep patterns during weekends or free days to cope with sleep debt accumulated during weekdays may be perceived as a justifiable behavioral choice for maintaining health and increasing cumulative sleep time. Among individuals younger than 65 years old, evidence suggests that long weekend sleep may compensate for the increased mortality risk associated with short weekend sleep [33]. However, recovery sleep during weekends does not appear to be an effective strategy to prevent metabolic dysregulation associated with recurrent insufficient sleep [34]. Our results provide further evidence that even the mild circadian misalignment associated with social jetlag may still be carcinogenic after taking account of sleep duration.

The biological mechanisms relating to circadian disruption and cancer development are complex and have been the focus of large-scale epidemiologic studies and controlled experimental studies, mostly in animal models [9,35]. The majority of accumulating evidence has focused on the effects of night shift work, which induces radical variations in exposure to light-dark and feeding cycles. This leads to misalignments between the circadian system and environmental cues as well as desynchronization among internal timing systems. Such discrepancies generate a profound influence on the temporal alignment of genetic and metabolic processes [36]. The carcinogenic effect of shift work has also been studied at the molecular level. For example, expression of the Period2 gene (*Per2*), a molecular component of circadian clocks, has been found to have tumor-suppressive effects [37]. *Per2* protein levels were found to be downregulated in proliferative prostate cancer cells compared to normal prostate cells, whereas melatonin treatment resulted in a resynchronization of oscillatory circadian rhythm genes (*Dbp* and *Per2*) [38]. To date, no studies have provided evidence of metabolic or molecular mechanisms that operate in the association between social jetlag and cancer development. There are several biological pathways through which social jetlag may influence prostate carcinogenesis (Figure S2). Social jetlag interacts with the sleep-wake cycle and contributes to circadian rhythm disruption. Circadian rhythm disruption could directly increase the risk of metabolic dysfunction such as obesity [15] and impaired circadian gene expression [39,40], both of which contribute to the initiation of molecular dysregulations that promote prostate carcinogenesis [39,41,42,43,44,45]. Circadian rhythm disruption may promote molecular dysregulation thus influence prostate cancer risk through a direct pathway, such as increased cell proliferation, increased sex steroid hormone, and insulin resistance [46]. While there may be similarities in the etiology, the influence of social jetlag may operate differently than other forms of sleep disturbance, such as night shift work. Importantly, social jetlag may not be associated with more exposure to light-at-night or sleep deprivation, both of which are posited as explanations of the carcinogenic effect of circadian disruption [7,29,47].

In the current study, stratified analyses by the onset of sleep time yielded elevated prostate cancer risk among subjects with early sleep preferences, based on a small number of incident cases. This is intriguing, as evening chronotype has previously been associated with an increased risk of prostate cancer in adults [48]. Our findings suggested that early chronotype may be more likely to be affected by circadian disruptions, which was also the hypothesis in a study reporting an elevated risk of type 2 diabetes with increased shift work duration among early chronotypes [49]. Future studies are warranted to replicate the observed social jetlag-prostate cancer relationship. In addition, longitudinal studies with a repeated measure on sleep patterns and highly controlled experimental studies are necessary to not only confirm this relationship but to also elucidate the biologic mechanisms by which social jetlag may increase the risk of prostate cancer. If the association between social jetlag and prostate cancer risk is deemed casual, future prostate cancer screening programs may consider the inclusion of social jetlag.

This study has several strengths. First, analyses were conducted in a large cohort study with over 8 years of follow-up, offering adequate power for the analyses of the social jetlag-prostate cancer relationship. No evidence to date has examined the social jetlag-prostate cancer risk based on a study of such size and length. Second, the study measured a range of sociodemographic characteristics (age, ethnicity, marital status, income, employment), behavioral factors (smoking [10,11,50,51] and alcohol status [52], physical activity and sedentary behavior [53,54,55,56], daily caloric consumption [57,58]) and physical conditions (body mass index [10,59], medical condition or comorbidity, and family history of cancer) that are potentially relevant to prostate cancer risk. An additional adjustment for sleep duration in the model to account for the potential effect of differing lengths of sleep [6]. Results from this model were similar to those from multivariate-adjusted models, offering compelling evidence.

Some limitations in this current study warrant discussion. First, although a number of covariates are included in the multivariable-adjusted analyses, some important confounders were not examined, such as the quality of sleep, the prevalence of sleep disorders, and the use of sleep medication. Second, the sleep data were measured using a self-reported questionnaire, which may cause misclassification of the exposure assessment, especially considering that poor recall is more likely to occur among individuals with sleep disorders [60]. Third, the sleep data were measured once in this study, which may not reflect the longitudinal changes in the population. Nevertheless, questions in our survey specifically asked for the “usual” sleep time, providing information on the habitual rather than a snapshot of sleep pattern. In addition, this measure may lead to a misclassification of social jetlag, which would have attenuated an association between social jetlag and prostate cancer risk and bias toward the null. However, a positive association was detected in the present analysis. Future studies employing device-based measures to monitor the sleep-wake cycles over a week or a monthly pattern that provide a robust measure of social jetlag to improve the exposure assessment is encouraged. Forth, chronotype was estimated using tertiles of mid-sleep time on free days, which may not reflect the true chronotype of the study participants but their chronotype tendency in the analyzed sample. Fifth, the data were mostly collected from educated White men with relatively high income who are more likely to get PSA testing, which may limit the generalizability of our results. Finally, despite that Alberta province has the highest prostate cancer incidence rate in Canada [61], the number of incident prostate cancer cases excluded the possibility of subgroup analyses by tumor aggressiveness. This should be explored in future population-based studies with sufficient cases of high-grade prostate cancer.

## 5. Conclusions

Our findings suggest that switching usual sleep patterns between weekday and weekend days among men is associated with an increased prostate cancer risk, especially in those with an early chronotype. Further population-based longitudinal studies are necessary to confirm these findings with sufficient cases of high-grade prostate cancer, and well-controlled experimental studies are necessary to elucidate the biological mechanism.

## Figures and Tables

**Table 1:** Descriptive statistics of the Alberta’s Tomorrow Project study population (*n* = 7455).

Participant Characteristics	Social Jetlag Category			
	0–<1 h	1–<2 h	2+ h	*p*-Values
Observations	3400	2204	1851	
Total person-years of observation	31,125.98	20,332.94	17,040.15	
Social jetlag (hours) ^1^	0.15 (0.24)	1.22 (0.27)	2.86 (1.37)	<0.001
Follow-up time (years) ^1^	9.15 (1.62)	9.23 (1.48)	9.21 (1.46)	0.208
Age (years) ^1^	58.77 (9.26)	53.44 (8.14)	51.86 (7.21)	<0.001
Sitting time (minutes/week) ^1^	19.30 (57.27)	20.81 (58.00)	23.04 (63.60)	0.092
Daily caloric consumption (kcal) ^1^	2164.79 (945.60)	2235.42 (964.35)	2273.67 (1001.16)	<0.001
Sleep duration (hours) ^1^	7.82 (1.16)	7.96 (0.99)	8.04 (1.07)	<0.001
Recreational PA (MET-hours/week) ^1^				0.011
Group 1 (0 h)	0.00 (0.00)	0.00 (0.00)	0.00 (0.00)	
Group 2 (>0.00–4.00 h)	2.42 (1.01)	2.46 (1.01)	2.42 (1.03)	
Group 3 (>4.00–8.25 h)	6.25 (1.37)	6.35 (1.29)	6.26 (1.27)	
Group 4 (>8.25–16.00 h)	12.01 (2.03)	12.17 (2.06)	11.97 (2.14)	
Group 5 (>16.00 h)	27.07 (11.03)	26.16 (8.91)	26.44 (9.14)	
Body mass index (kg/m^2^) ^2^				0.540
Normal (18.5–<25)	737 (21.7)	515 (23.3)	396 (21.4)	
Overweight (25–<30)	1662 (48.9)	1086 (49.3)	922 (49.8)	
Obese (30+)	991 (29.1)	599 (27.2)	528 (28.5)	
Missing	10 (0.3)	4 (0.2)	5 (0.3)	
Marital status ^2^				0.002
Married, or living with someone	2846 (83.7)	1902 (86.3)	1524 (82.3)	
Divorced, separated, or widowed	352 (10.4)	205 (9.3)	197 (10.7)	
Single, never married	202 (5.9)	97 (4.4)	130 (7.0)	
Education ^2^				<0.001
Some or all post-secondary	2456 (72.2)	1757 (79.7)	1475 (79.7)	
High school or less	944 (27.8)	447 (20.3)	376 (20.3)	
Employment status ^2^				<0.001
Currently employed	2008 (59.1)	1907 (86.5)	1754 (94.8)	
Not currently employed	1392 (40.9)	297 (13.5)	97 (5.2)	
Total household income ^2^				<0.001
$0–$49,999	842 (24.8)	266 (12.0)	191 (10.3)	
$50,000–$99,999	1259 (37.0)	733 (33.3)	637 (34.4)	
$100,000+	1276 (37.5)	1198 (54.4)	1014 (54.8)	
Missing	23 (0.7)	7 (0.3)	9 (0.5)	
Smoking status ^2^				<0.001
Never	1390 (40.9)	1029 (46.7)	798 (43.1)	
Former	1475 (43.4)	818 (37.1)	704 (38.0)	
Current	533 (15.7)	357 (16.2)	349 (18.9)	
Missing	2 (0.1)	0 (0.0)	0 (0.0)	
Frequency of alcohol consumption ^2^				<0.001
Never	431 (12.7)	213 (9.7)	185 (10.0)	
Rarely	606 (17.8)	381 (17.3)	332 (17.9)	
Occasional	1160 (34.1)	856 (38.28)	707 (38.2)	
Regular	316 (9.3)	176 (8.0)	127 (6.9)	
Missing	887 (26.1)	578 (26.2)	500 (27.0)	
Family history of cancer ^2^				0.539
Yes	2454 (72.2)	1632 (74.1)	1363 (73.6)	
No	939 (27.6)	569 (25.8)	484 (26.2)	
Missing	7 (0.2)	3 (0.1)	4 (0.2)	
Pre-existence of medical conditions ^2^				<0.001
Yes	829 (24.4)	698 (31.7)	599 (32.4)	
No	2571 (75.6)	1506 (68.3)	1252 (67.6)	
PSA (prostate-specific antigen) screening ^2^				
Yes	1899 (55.9)	1053 (47.8)	801 (43.3)	
No	1501 (44.1)	1151 (52.2)	1050 (56.7)	
Chronotype ^2^				<0.001
Early	2085 (61.3)	790 (35.8)	327 (17.7)	
Intermediate	797 (23.5)	795 (36.1)	547 (29.5)	
Late	518 (15.2)	619 (28.1)	976 (52.7)	
Missing	0 (0.0)	0 (0.0)	1 (0.1)	

^1^ Denoted values: mean (standard deviation). ^2^ Denoted values: frequency (proportion of column total).

**Table 2:** Hazard ratio estimates of prostate cancer incidence by social jetlag and chronotype in the Alberta’s Tomorrow Project study.

Social Jetlag	Age-Adjusted Model		Multivariate-Adjusted Model ^1^		Multivariate-Adjusted Model ^2^		Multivariate-Adjusted Model ^3^	
	Cases	HR (95% CI)	Cases	HR (95% CI)	Cases	HR (95% CI)	Cases	HR (95% CI)
All chronotypes included								
0–<1 h	117	1.00 (Reference value)	115	1.00 (Ref.)	115	1.00 (Ref.)	114	1.00 (Ref.)
1–<2 h	75	1.48 (1.09 to 2.00)	74	1.52 (1.10 to 2.01)	74	1.49 (1.09 to 2.06)	72	1.45 (1.05 to 2.01)
2+ h	58	1.58 (1.12 to 2.22)	58	1.69 (1.15 to 2.46)	58	1.64 (1.12 to 2.41)	55	1.54 (1.04 to 2.27)
*p* for trend	-	0.004	-	0.004	-	0.006	-	0.018
Early chronotypes only								
0–<1 h	73	1.00 (Ref.)	72	1.00 (Ref.)	72	1.00 (Ref.)	71	1.00 (Ref.)
1–<2 h	33	1.66 (1.08 to 2.54)	33	1.73 (1.12 to 2.68)	33	1.75 (1.12 to 2.72)	33	1.76 (1.13 to 2.75)
2+ h	15	1.98 (1.11 to 3.52)	15	2.10 (1.16 to 3.77)	15	2.12 (1.18 to 3.83)	14	2.04 (1.11 to 3.74)
*p* for trend	-	0.004	-	0.003	-	0.003	-	0.004
Intermediate chronotypes only								
0–<1 h	22	1.00 (Ref.)	22	1.00 (Ref.)	22	1.00 (Ref.)	22	1.00 (Ref.)
1–<2 h	23	1.51 (0.81 to 2.80)	23	1.43 (0.74 to 2.73)	23	1.35 (0.71 to 2.58)	21	1.20 (0.62 to 2.33)
2+ h	17	1.87 (0.94 to 3.75)	17	1.72 (0.82 to 3.62)	17	1.53 (0.72 to 3.22)	15	1.29 (0.59 to 2.80)
*p* for trend	-	0.071	-	0.150	-	0.263	-	0.518
Late chronotypes only								
0–<1 h	22	1.00 (Ref.)	21	1.00 (Ref.)	21	1.00 (Ref.)	21	1.00 (Ref.)
1–<2 h	19	1.24 (0.65 to 2.34)	18	1.30 (0.65 to 2.61)	18	1.28 (0.64 to 2.57)	18	1.32 (0.65 to 2.68)
2+ h	26	1.28 (0.69 to 2.39)	26	1.39 (0.69 to 2.80)	26	1.35 (0.70 to 2.74)	26	1.38 (0.68 to 2.81)
*p* for trend	-	0.441	-	0.381	-	0.416	-	0.392

^1^ Adjusted for age, marital status, highest level of completed education, total household income, employment status, smoking status, frequency of alcohol consumption, recreational physical activity, total sitting time, pre-existence of medical conditions, and chronotype (all chronotype sub-table only). ^2^ Further adjusted for sleep duration. ^3^ Further adjusted for body mass index, daily caloric consumption, family history of cancer, and PSA screening.

## Supplementary Materials

The following are available online at https://www.mdpi.com/2072-6694/12/12/3873/s1, Figure S1: The directed acyclic graph (DAG) to identify potential confounders a priori in the prospective association between social jetlag and prostate cancer incidence in the Alberta’s Tomorrow Project, Figure S2: Hypothesized pathway through which social jetlag associated sleep-wake cycle disruption may influence prostate cancer risk.
