# Supplementary material for: Social Jetlag and Prostate Cancer Incidence in Alberta’s Tomorrow Project: A Prospective Cohort Study
Source: Cancers (Basel). 2020 Dec 21;12(12):3873. doi: 10.3390/cancers12123873 (PMC7767515; doi:10.3390/cancers12123873)
Supplement: Supplementary file 1 [file cancers-12-03873-s001.pdf]

Supplementary Material: Social Jetlag and Prostate Cancer Incidence in Alberta's Tomorrow Project: A Prospective Cohort Study

Liang Hu, Andrew Harper, Emily Heer, Jessica McNeil, Chao Cao, Yikyung Park, Kevin Martell, Geoffrey Gotto, Grace Shen-Tu, Cheryl Peters, Darren Brenner and Lin Yang

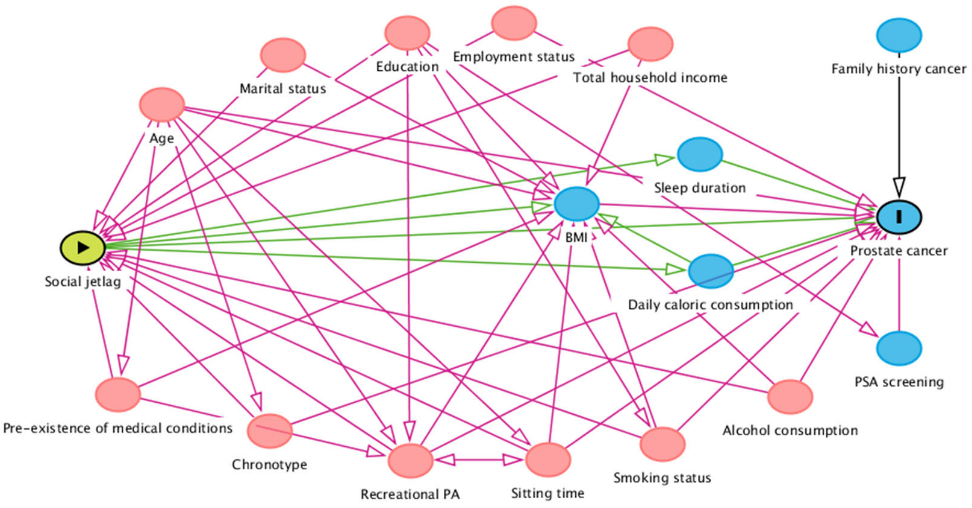

Figure S1. The directed acyclic graph (DAG) to identify potential confounders a priori in the prospective association between social jetlag and prostate cancer incidence in the Alberta's Tomorrow Project.

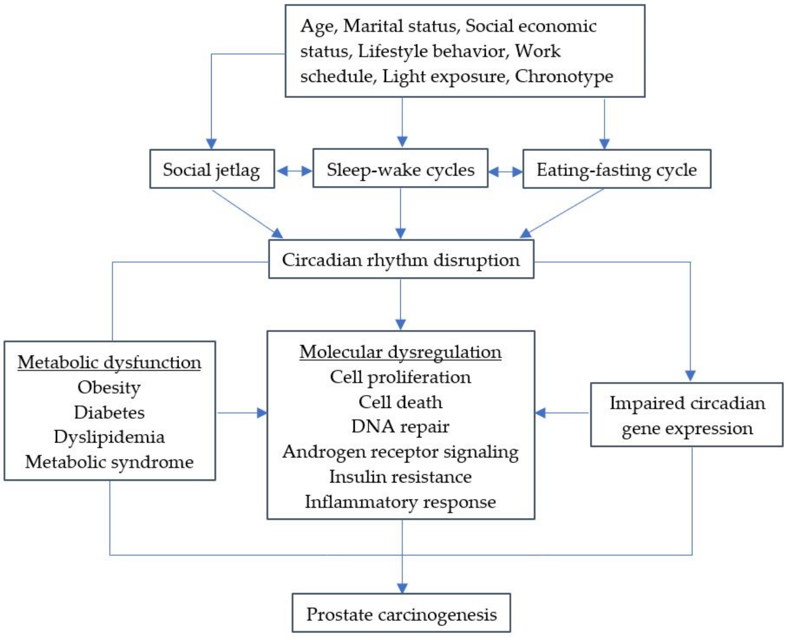

Figure S2. Hypothesized pathway through which social jetlag associated sleep-wake cycle disruption may influence prostate cancer risk.
